# Optimal management after paediatric lumbar puncture: a randomized controlled trial

**DOI:** 10.1186/s12883-019-1275-9

**Published:** 2019-04-13

**Authors:** Bing Hu, Tian-ming Chen, Bing Liu, Wei Chi, Yi-qing Miao, Xiao-lu Nie, Xiao-xia Peng, Gang Liu

**Affiliations:** 1 Department of Infectious Diseases, Beijing Children’s Hospital, Capital Medical University, Nalishi Road 56#, Xicheng District, Beijing, 100045 China; 2 Center for Clinical Epidemiology and Evidence-based Medicine, Beijing Children’s Hospital, Capital Medical University, Nalishi Road 56#, Xicheng District, Beijing, 100045 China

**Keywords:** Lumbar puncture, Duration of bed rest, Supine position, Postoperative complications

## Abstract

**Background:**

To evaluate whether a shorter time of lying supine without a pillow and fasting for solids and liquids (LSFSL) after a lumbar puncture (LP) is associated with a higher risk of post-lumbar puncture headache (PLPH) and post-lumbar puncture lower back pain (PLPBP) in a randomized, assessor-blinded, controlled trial.

**Methods:**

Paediatric patients who underwent their first LP after hospital admission were randomly allocated to either the group with half an hour of LSFSL (0.5 h LSFSL) or 4 h of LSFSL (4 h LSFSL) immediately after LP. The primary outcome is PLPH after LP. The incidence of PLPH, PLPBP, and vomiting; vital signs (respiratory rate, heart rate, blood pressure); and other post-procedure conditions after LP were measured as the outcomes. The Non-inferiority test and Wilcoxon rank-sum test were used to analyse the outcome data.

**Results:**

In total, 400 patients (201 in the 0.5-h LSFSL group and 199 in the 4-h LSFSL group) were included in this trial. Twelve (5.97%) of 201 patients experienced PLPH in the 0.5 h LSFSL group versus 13 (6.53%) of 199 patients in the 4 h LSFSL group (difference 0.56, 95% CI -4.18 to 5.31; *p* = 0·0108 for the non-inferiority test). Fourteen (6.97%) of 201 patients experienced PLPBP in the 0.5 h LSFSL group versus 17 (8.54%) of 199 patients in the 4 h LSFSL group (difference 1.57, 95% CI -3.66 to 6.82; *p* = 0.007 for the non-inferiority test). The changes in heart rate (HR), respiratory rate (RP) and systolic blood pressure (SBP) before and after the LP were not different between the 0.5-h LSFSL group and the 4-h LSFSL group. No other adverse events were reported.

**Conclusions:**

Compared with 4 h of LSFSL after LP, 0.5 h of LSFSL was not associated with a higher risk of PLPH, PLPBP or other adverse events. In conclusion, 0.5 h of LSFSL is sufficient for children undergoing LP.

**Trial registration:**

Clinical trial NCT02590718. The date of registration was 08/25/2015.

## Background

Lumbar puncture (LP) is a common clinical procedure that is widely used in the diagnosis of central nervous system infections, intracranial autoimmune diseases, subarachnoid haemorrhage, and intracranial tumours. Post-lumbar puncture headache (PLPH) and post-lumbar puncture lower back pain (PLPBP) are the most common post-procedure complications [1]. PLPH is a condition thought to result from failure of the dural puncture site to close, leading to cerebrospinal fluid (CSF) leakage and intracranial hypovolemia. This causes traction on the pain-sensitive structures in the brain, resulting in a headache [2, 3]. Other factors correlated with the occurrence of PLPH include age, sex, body mass index, the needle gauge, the shape of the needle point, the needle orientation, the direction of the needle bevel, withdrawal of the needle core, and the operator skill level [4, 5]. In domestic and foreign studies, no significant correlation was found between the duration of bed rest in the supine position without a pillow immediately after the LP and the occurrence of post- procedure complications, including PLPH. Due to the lack of a standard duration of bed rest after LP in China, most patients are advised to lie down for 4–6 h. It can be challenging to obtain cooperation with bed rest from paediatric patients for that length of time. In the meanwhile, fasting for solids and liquids may cause low blood sugar, leading to complications and inconvenience for both patients and doctors. In this randomized controlled clinical trial, we studied whether the duration of bed rest in the supine position without a pillow while fasting for solids and liquids (LSFSL) is associated with PLPH and PLPBP.

## Methods

### Setting

This research was conducted at the Infectious Disease Department of Beijing Children’s Hospital between March 2015 and December 2017. It was approved (No. 2015–2) by the Ethics Committee of Beijing Children’s Hospital affiliated with Capital Medical University. The protocol (ID: 20150826) was registered in ClinicalTrials.gov.

### Participants

We recruited participants undergoing their first LP after admission. The inclusion criteria were as follows: 29 days to 18 years old, normal mental status, and no contraindications for LP. The exclusion criteria were as follows: contraindication for LP, mental retardation or mental symptoms, incapable of mobilization after LP, signs of unconsciousness, lidocaine allergy, history of headache and lower back pain before LP, prior LP.

### Randomization and blinding

An independent statistician prepared randomization numbers using a computer-generated random assignment procedure. The results of the randomization allocation were kept in envelopes. Each envelope was designated for one participant. The envelopes were opened when he participants joined the trial. All participants were informed that there was a 50% chance of being assigned to the 0.5-h LSFSL group or the 4-h LSFSL group. The data were analysed by an independent statistician who was blinded to the participants’ allocation.

### Interventions

All LPs were performed by two experienced clinicians. To avoid possible bias caused by practitioner interaction, equal numbers of participants who were assigned to the 0.5-h and 4-h LSFSL groups were randomly allocated to each clinician. The LP procedures strictly followed the instructions in *Zhu Fu-tang Practical Paediatrics (8th ed.)* [6]. A standard LP package with the 22 gauge puncture needle was used for patients all the patients. All the patients had no pre-procedure sedation. All the patients were placed in a lateral knee-chest position, and the needle was inserted parallel to the floor with the bevel pointing upwards; 3–10 ml of CSF was collected, the needle core was inserted, and the needle was withdrawn. Patients then underwent 0.5 h or 4 h of LSFSL after the LP, according to their assigned group.

### Outcomes

A study nurse in the ward observed the patients for symptoms of headache, lower back pain, nausea and cerebral hernia during the 5 days after the LP. When symptoms occurred, the time and treatment were recorded. Our nurse assessed the patients from 4 h to 5 days after LP every 8 h every day. If the patients have discomfort complaint at any time, we will also make an assessment immediately. Patients less than 2 years old can’t describe their pain. The Face, Legs, Activity, Cry, and Consolability (FLACC) behavioral pain scale was previously found to have excellent validity and reliability for pain assessment in young, cognitively intact children [7]. So we used FLACC to access the patients less than 2 years old whether they had pain or not. Our study nurse observed these patients for at least 1–2 min from 4 h to 5 days after LP every 8 h every day. Vitals were monitored hourly for 4 h after the LP. The results are presented in the CRF table. All the doctors and the study nurse were specially trained before participating in this study. For the diagnosis of PLPH, we followed the guidelines of *The International Headache Society* and the *International Classification of Headache (ICHD-II)*. PLPH was defined as a headache occurring within 5 days of the LP and disappearing spontaneously within a week or within 48 h of the successful treatment of CSF leakage; PLPHs intensify after 15 min of sitting or standing up from a recumbent position and remits within 15 min of lying down. The definition of PLPBP was persistent pain surrounding the location of the puncture [4, 8]. Patients’ heart rates (HR), respiratory rates (RR), and systolic blood pressure (SBP) before and after the LP were also recorded.

### Withdrawal from the study

Participants were free to withdraw at any time without having to provide a reason during the entire trial period.

### Adverse events

Participants were instructed to record any unexpected signs or symptoms in CRFs during the trial period. All participating doctors and study nurses were required to record any observed adverse events (AEs).

### Statistical methods

The primary outcomes were analysed by non-inferiority test (Confidence interval method) and The changes in HR, RP and SBP before and after the LP between two groups were analysed by Student’s t test. The baseline binary variable of sex was compared by the χ^2^ test and other baseline continuous variables were compared by Wilcoxon test if the normal distribution were not satisfied. If *p* value was < 0.05, the differences were statistically significant except for non-inferiority test, which the significance level of the test was 0.025. All statistical analyses were conducted using SPSS 17.0 (SPSS Inc., Chicago, IL, USA) and JMP 14.0.

### Sample size calculation

PASS 16.0 was used to calculate the sample sizes. Sample sizes of 171 in 0.5 h LSFSL group and 171 in 4 h LSFSL achieved 80.07% power to detect a non-inferiority margin difference between the group proportions of 0.05.The reference group (4 h LSFSL) proportion of headache was 15% while the treatment group (0.5 h LSFSL) proportion was 20% under the null hypothesis of inferiority. The power was computed for the case when the actual treatment group proportion was 10%. The test statistic used is the one-sided Z test. The significance level of the test was 0.025. We intended to recruit 200 participants for each group to allow for a rate of loss to follow-up of 10%.

## Results

### Participants and baseline characteristics

**Fig. 1:**
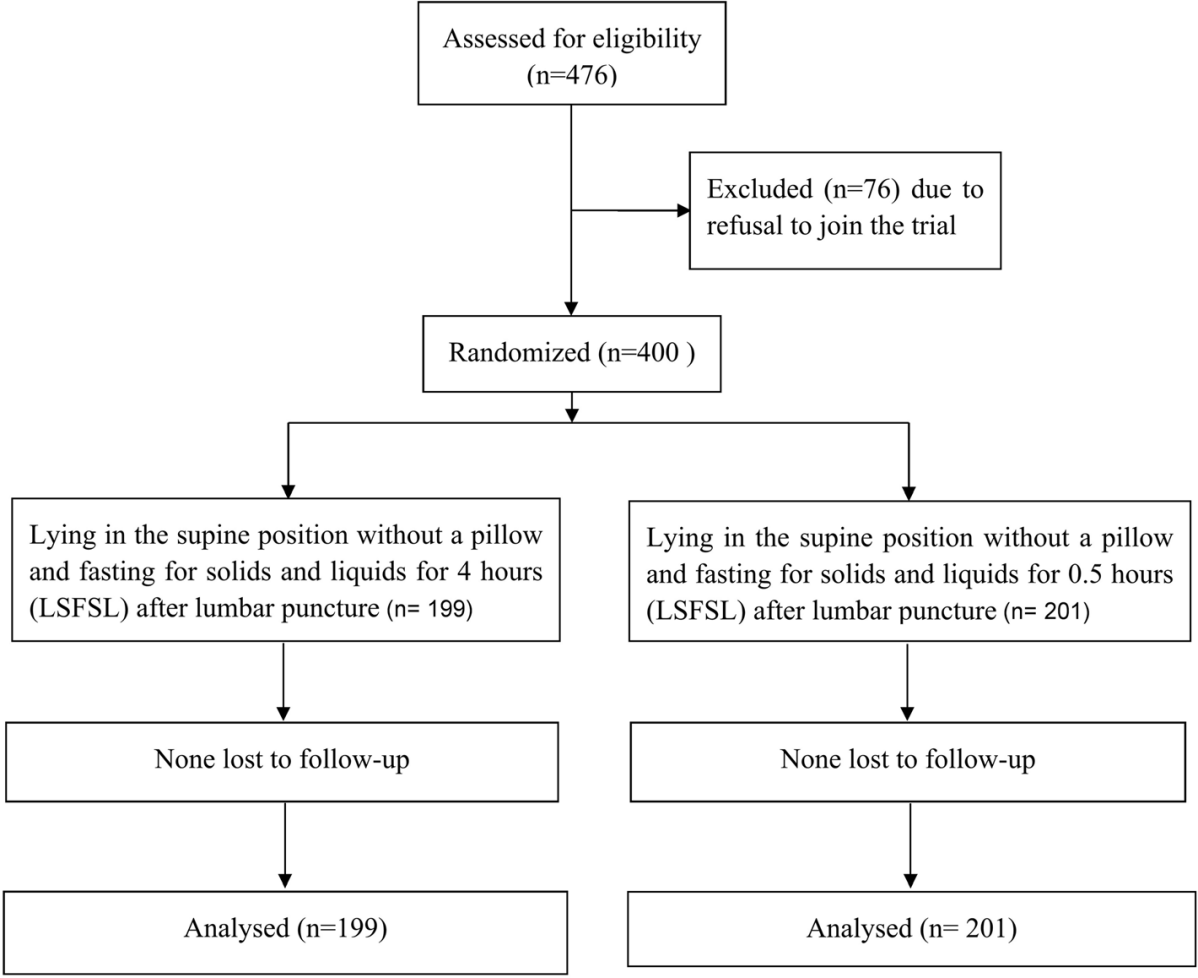
Flow diagram of the trial procedure. 0.5-h LSFSL: lying in the supine position without a pillow and fasting for solids and liquids for half an hour after lumbar puncture; 4-h LSFSL: lying in the supine position without a pillow and fasting for solids and liquids for 4 h after lumbar puncture; HR: heart rate; RR: respiratory rate; SBP: systolic blood pressure

In total, 400 inpatients were enrolled. They were randomized to the 0.5-h LSFSL group (*n* = 201) and the 4-h LSFSL group (*n* = 199). A flow diagram of the trial procedure is shown in Fig. 1. CSF was successfully collected from all 400 patients, which is an LP success rate of 100%; 92% of patients (368 patients) were one attempt. Other 8% (32 patients) were 2–4 attempts.

There were no significant differences between the 2 groups with regard to the participants’ age, sex, or amount of CSF collected. All patient demographics were comparable between the two groups. The results are shown in Table 1. The vital signs (respiratory rate, pulse, heart rate, blood pressure) of all patients were within the normal ranges before they underwent the LP.

**Table 1:** Demographic characteristics of the included participants

	0.5 h LSFSL (*n* = 201)	4 h LSFSL (*n* = 199)	test value	P
Sex			3.242^*^	0.073
Male, n(%)	120(59.7)	136(68.3)		
Female, n(%)	81(40.3)	63(31.7)		
Age(y), median [IQR]	1 .0(0.3–6.0)	2.0 (0.3–6.0)	0.191^#^	0.658
Vital signs before LP				
RR(per minute), median [IQR]	26(22–31)	26(22–31)	0.269^#^	0.604
HR(per minute), median [IQR]	120(98–132)	120(100–135)	0.293^#^	0.588
SBP(mmHg), median [IQR]	90(80–96.5)	90(80–96)	0.057^#^	0.810
Number of attempts, median [IQR]	1(1–1)	1(1–1)	0.002^#^	0.963
Amount of CSF(ml), median [IQR]	5(3–5)	5(3–5)	1.021^#^	0.312

*LSFSL* lying in the supine position without a pillow and fasting for solids and liquids, *HR* heart rate, *RR* respiratory rate, *SBP* systolic blood pressure, *CSF* cerebral spine fluid

^*^t value

^#^z value

### Outcomes and estimation

The FLACC scores of the patients who were under 2 years old show no pain from 4 h to 5 days after LP. So all the patients younger than 2 years didn’t suffer PLPH and PLPBP. The rate of PLPH was 5.97% (95% CI 3.12 to 10.2) in 0.5 h LSFSL group and 6.53% (95% CI 3.52 to 10.9) in 4 h LSFSL group. The one-sided 95% CI for the underpowered non-inferiority test on the rate difference was 0.56% (95% CI -4.18 to 5.31), with a non-inferiority margin of 5%. The results are shown in Table 2.

**Table 2:** Incidence of headache or back pain after lumber puncture

Events	0.5 h LSFSL (*n* = 201)	4 h LSFSL (*n* = 199)	Z value	*P* value	Non-inferiority margin	Rate difference	95% confidence interval
PLPH, n(%)	12(5.97%)	13(6.53%)	2.298	0.011	−0.05	0.0056	−0.0418 to 0.0531
PLPBP, n (%)	14(6.97%)	17(8.54%)	2.460	0.007	−0.05	0.0157	−0.0366 to 0.0682
Composite endpoint, n(%)	24(11.94%)	25(12.56%)	1.715	0.043	−0.05	0.0062	−0.058 to 0.0705

*LSFSL* lying in the supine position without pillow and fasting for solids and liquids, *PLPBP* post lumbar puncture lower back pain, *PLPH* post lumbar puncture headache

The rate of PLPBP was 6.97% (95% CI 3.86 to 11.4) in 0.5 h LSFSL group and 8.54% (95% CI 5.06 to 13.3) in 4 h LSFSL group. The one-sided 95% CI for the underpowered non-inferiority test on the rate difference was 1.57% (95% CI -3.66 to 6.82), with a non-inferiority margin of 5%. The results are shown in Table 2.

**Fig. 2:**
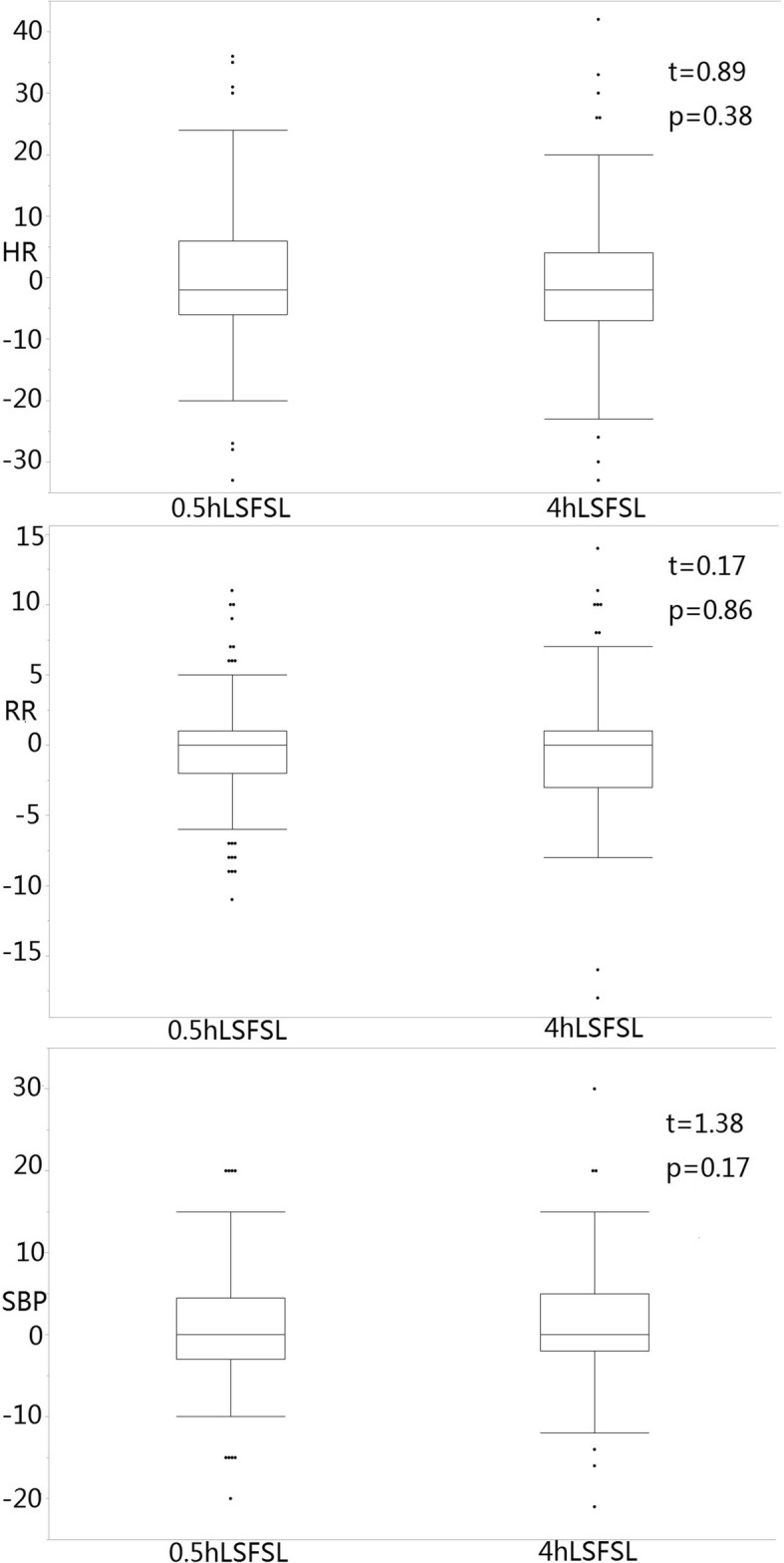
Mean differences in other physiological indexes after lumber puncture

All patients’ vital signs (respiratory rate, pulse, heart rate, blood pressure) were within the normal ranges 4 h after they underwent the LP. The changes in HR, RP and SBP before and after the LP were − 0.144 ± 10.641 (per minute), − 0.408 ± 3.493 (per minute), 0.503 ± 6.241 (mmHg) respectively in 0.5 h LSFSL group and − 1.070 ± 10.214 (per minute), − 0.347 ± 3.755 (per minute), 1.397 ± 6.692 (mmHg) respectively in 4 h LSFSL group. The changes in HR, RP and SBP before and after the LP were not significantly different between the 0.5-h and 4-h LSFSL groups. The results are shown in Fig. 2. No other AEs were reported during the trial.

## Discussion

Our research suggests that in paediatric patients, LSFSL for 30 min or 4 h result in equivalent incidence rates of PLPH and PLPBP, and the duration of LSFSL does not affect the patient’s vital signs. However, prolonged LSFSL can be inconvenient for both the patients and the medical staff. Standardized instructions should be strictly followed to improve the LP procedure in children.

LP is a basic clinical procedure that is widely used in paediatric medicine. The procedure is safe when the instructions are strictly followed. However, complications can occur, and PLPH is the most common complication [1]. The incidence of PLPH varies in domestic and foreign research papers. Ren Bo analysed 198 children who underwent LP and reported a PLPH incidence rate of 5.05% and a PLPBP incidence rate of 11.62% [9]. This result is similar to ours. In foreign research papers, the incidence of PLPH was found to be approximately 40% (1–70%) [10], and according to a retrospective analysis of foreign research papers by Yen-Feng Wang [3], the incidence of PLPH reached approximately 1/3 of patients undergoing LP.

It is believed that PLPH is caused by intracranial hypotension due to the leakage of CSF after LP [3, 4]. According to the review by Yan Wei Ling, PLPH has no significant correlation with either age or sex. A smaller amount of collected CSF (< 15 ml) and the number of previous LPs had also no significant correlations with PLPH [11]. Other factors correlated with PLPH include the following: needle gauge, the shape of the needle point, the needle orientation, the direction of the needle bevel, withdrawal of the needle core, and operator skill level [4, 5]. It has been observed that the model of needle used significantly affects the incidence of PLPH. The larger the needle, the higher the incidence rate of PLPH is [8, 12, 13]. In China, the No.7 puncture needle (0.7 mm in diameter) with a lead bevel is the common choice for LP. For neonates and infants, the No. 5.5 puncture needle (0.55 mm in diameter) has been recommended by previous studies, as it increases the success rate of LP and reduces haemorrhage, with the only disadvantage being the loss of the ability to measure the cerebrospinal fluid pressure (CSFP) [14]. In our study, we standardized the factors influencing PLPH by using a standard LP package with 22 gauge puncture needle, arranging for clinicians of the same skill level to perform the LPs, using the same puncture angle with the bevel facing upwards and always withdrawing the needle with the core inside. Also the amount of CSF collected from each participant ranged from 3 to 10 ml. The results showed a PLPH incidence rate of 6.25% and a PLPBP incidence rate of 7.75%; no instances of cerebral hernia or other severe complications occurred during the research. These results are in agreement with the statistics reported in domestic and foreign research papers.

At present, the treatment for PLPH is LSFSL immediately after the LP, but there is no standard management guide for post-LP patients. In clinical practice, patients are routinely advised to LSFSL for 4–6 h after the LP to prevent PLPH and PLPBP [15]. According to *Zhu Fu-tang Practical Paediatrics (8th ed.)*, the patient should lie supine position without a pillow for 30–60 min after a LP, and fasting is not mentioned. After a reviewing domestic and foreign studies, we found no evidence that supports the idea that a longer duration of bed rest reduces the risk of PLPH [16–20]. In contrast, a study has showed that a longer duration of bed rest could be related to a higher incidence of PLPH. In China, Jin Xiao Ping’s research on adult LPs and Yan Wei Ling’s study on paediatric LPs found no significant correlation between the duration of post-LP bed rest and the incidence of PLPH [11, 21]. In agreement with the studies above, our study revealed that 0.5 h of LSFSL was not associated with a higher risk of PLPH, PLPBP or other adverse events, compared with 4 h of LSFSL after LP. This finding demonstrated that the duration of LSFSL was not correlated with the incidence of PLPH. Since it is difficult for paediatric patients, especially for infants, to cooperate with remaining still in the supine position without a pillow for hours, long periods of post-LP bed rest has often been an inconvenience for both the patients and the medical staff. The results of this research indicate that in the future, it is only necessary to maintain 30 min of LSFSL after the LP, instead of 4–6 h.

In this research, the enrolled patients younger than 2 years old can’t clearly describe headache or lower back ache. This might be a limitation, but FLACC score can be used to assess pain from burns and other etiologies for preverbal children and was previously found to have excellent validity and reliability for pain assessment in young, cognitively intact children. So our result is reliable to a great extent.

## Conclusions

In conclusion, compared with 4 h of LSFSL after LP, 0.5 h of LSFSL is not associated with higher incidence rates of PLPH, PLPBP or other adverse event. In conclusion, 0.5 h of LSFSL is adequate for children who have undergone a LP.

## Abbreviations

0.5-h LSFSL: Lying in the supine position without a pillow and fasting for solids and liquids for half an hour after lumbar puncture
4-h LSFSL: Lying in the supine position without a pillow and fasting for solids and liquids for four hours after lumbar puncture
HR: Heart rate
LP: Lumbar puncture
LSFSL: Lying in the supine position without a pillow and fasting for solids and liquids
PLPBP: Post-lumbar puncture lower back pain
PLPH: Post-lumbar puncture headache
RR: Respiratory rate
SBP: systolic blood pressure
